# Global trends in carbapenem-resistant gram-negative bacteria research (2020–2025): a bibliometric analysis and systematic review

**DOI:** 10.3389/fcimb.2025.1690417

**Published:** 2025-12-10

**Authors:** Xiaotong Zhang, Ce Zhang, Mengyao Lv, Shu Wang, Qiuting Wang, Chengshuai Yang, Qian Zhao, Luyuan Ma, Bei Li, Xiaohua Qin, Caiyan Zhao, Chuan Shen

**Affiliations:** 1 Department of Infectious Disease, Hebei Medical University Third Hospital, Shijiazhuang, China; 2 Clinical Research Center for Infectious Disease of Hebei Province, Shijiazhuang, China; 3 Hebei Key Laboratory for Diagnosis, Treatment, Emergency Prevention and Control of Critical Infectious Diseases, Shijiazhuang, China; 4 Office of Quality Management and Control in Healthcare, Hebei Medical University Third Hospital, Shijiazhuang, China; 5 Department of Infectious Disease, Qinghe People’s Hospital, Xingtai, China; 6 Institute of Antibiotics, Huashan Hospital of Fudan University, Shanghai, China

**Keywords:** CRGNB, carbapenem-resistant *Klebsiella pneumoniae*, *Escherichia coli*, carbapenemases, efflux pumps, porin mutations, antibiotic resistance, β-lactam/β-lactamase inhibitors

## Abstract

**Background:**

Carbapenem-resistant Gram-negative bacteria (CRGNB) pose a severe global health threat, yet comprehensive bibliometric analyses in this field remain limited. This systematic review employs a bibliometric methodology to identify research hotspots and emerging trends from 2020 to 2025.

**Method:**

Literature published between January 1, 2020, and October 31, 2025, was retrieved from the Web of Science Core Collection (WoSCC), Scopus, and PubMed for bibliometric analysis. Analytical tools, including VOSviewer, CiteSpace, and the Bibliometrix package, were used to assess publications by number, country, institution, journal, author, and keywords.

**Results:**

The bibliometric analysis revealed that global CRGNB research has experienced a fluctuating growth trend. China was the leading contributor, with 2,950 publications (25.5% of the total), and demonstrated significant collaboration with the USA and the UK. Major research clusters encompassed hypervirulent CRGNB strains (particularly carbapenem-resistant *Klebsiella pneumoniae* and *Escherichia coli*), resistance mechanisms (particularly carbapenemase-producing), antibiotic resistance, emerging therapeutic strategies (such as novel β-lactam/β-lactamase inhibitors, siderophore antibiotics, phage therapy, and antimicrobial peptides) and One Health perspectives (addressing environmental reservoirs). Thematic analysis identified evolving research priorities, including hypervirulent CRGNB strains, artificial intelligence, and early diagnosis and rapid screening of carbapenem resistance, exemplified by clustered regularly interspaced short palindromic repeats-based detection and artificial intelligence-driven matrix assisted laser desorption ionization-time of flight analysis. Randomized controlled trials indicated promising outcomes for several new antimicrobial agents, such as cefiderocol, sulbactam-durlobactam, and imipenem-relebactam. However, safety concerns, particularly in critically ill patients, remain a significant challenge.

**Conclusion:**

CRGNB research is increasingly directed toward elucidating resistance mechanisms, improving diagnostic tools, and exploring non-antibiotic therapeutic options. Strengthening international collaboration and fostering multidisciplinary approaches are imperative to advance high-quality research and address this growing threat.

## 1 Introduction

Antimicrobial resistance (AMR) has emerged as a critical global health crisis (Salam et al., 2023; GBD 2021 Antimicrobial Resistance Collaborators, 2024), with the global and regional forecasts of 1.91 million deaths attributable to AMR and 8.22 million fatalities associated with AMR in 2050 (GBD 2021 Antimicrobial Resistance Collaborators, 2024). The rapid dissemination of antibiotic-resistant strains within healthcare settings has led to an increase in difficult-to-treat infections, often resulting in prolonged hospital stays, increased healthcare costs, and higher mortality rates (Oliveira et al., 2024; Ho et al., 2025). The prevalence of carbapenem-resistant Gram-negative bacteria (CRGNB) continues to increase, the highest documented *in vitro* isolation rates of CRGNB and the most severe clinical outcomes—such as increased mortality and morbidity—are observed in carbapenem-resistant *Enterobacteriaceae* (CRE), carbapenem-resistant *Acinetobacter baumannii* (CRAB), and carbapenem-resistant *Pseudomonas aeruginosa* (CRPA), representing major public health threats (Mancuso et al., 2023). Recognizing their severe threat, the World Health Organization (WHO) designated CRGNB as “critical” and “high” priority groups in its 2024 bacterial priority pathogens list (World Health Organization, 2024a). These developments have prompted further scientific investigation and collaboration into CRGNB worldwide.

CRGNB strains are generally categorized as extensively drug-resistant (XDR) or pan-drug-resistant (PDR), reflecting their resistance to a wide range of currently available antibiotics (Zeng et al., 2023). This therapeutic crisis primarily results from both the limited availability of effective treatment options and the remarkable adaptive capacity of these pathogens (Jean et al., 2022). Surveillance data from the China Antimicrobial Surveillance Network (CHINET) indicate that CRGNB strains, particularly carbapenem-resistant *Escherichia coli* (CREC), carbapenem-resistant *Klebsiella pneumoniae* (CRKP), CRAB, and CRPA, now account for a substantial proportion of clinical isolates, with prevalence rates demonstrating a growing trend (Qin et al., 2024). Current research efforts are focused on elucidating the molecular mechanisms underlying CRGNB resistance and identifying novel therapeutic strategies (Nino-Vega et al., 2025; Aurilio et al., 2022; Basaran and Oksuz, 2025). However, the development of new antibiotics has not kept pace with the rapid evolution of drug-resistant strains. Moreover, the pipeline for new antibiotics remains extremely limited and insufficient to address the rapid evolution of carbapenem resistance mechanisms (Macesic et al., 2025; Muteeb et al., 2023). Furthermore, the clinical efficacy of existing antimicrobials is diminishing as CRGNB develop increasingly sophisticated resistance mechanisms; this phenomenon has prompted many pharmaceutical companies to scale back or even withdraw from antibiotic research (Salam et al., 2023). Recently, non-antibiotic therapies, such as bacteriophage applications and immunomodulatory strategies, have emerged as potential alternatives, but their clinical implementation faces substantial challenges (Kumar et al., 2021; Hickson et al., 2025). These challenges are further exacerbated by systemic barriers to accessing antimicrobial drugs, particularly regulatory hurdles in developing countries that delay approvals of new antibiotics. Despite global efforts to contain the problem, CRGNB infections continue to rise relentlessly, underscoring the urgent need for innovative therapies and stronger international collaboration.

Bibliometric analysis is defined as a quantitative research methodology that employs mathematical and statistical techniques to summarize, analyze, and quantify data from scientific literature (Manoj Kumar et al., 2023). By generating knowledge maps, this approach provides a comprehensive overview of developmental trajectories, research hotspots, and emerging trends within a field (Du et al., 2024; Sun et al., 2024). In recent years, bibliometric analysis has been widely utilized to analyze academic publications, identifying influential countries, journals, institutions, and authors, as well as key publications, references, and keywords (Sun et al., 2024). The analysis tools such as CiteSpace and VOSviewer are commonly used to visualize collaboration networks between countries, institutions and authors, thereby revealing the dynamics of scientific cooperation (Du et al., 2024). Although there are relevant quantitative analyses on individual CRGNBs, such as CRE and CRAB, a comprehensive analysis of the prevailing research trends and key topics in this domain remains notably absent. Therefore, we aim to perform a comprehensive bibliometric analysisto systematically delineate current research trends, major themes, and future directions in the field of CRGNB.

## 2 Materials and methods

### 2.1 Literature sources and search strategies

This systematic review incorporated a bibliometric analysis of global CRGNB research trends. Our bibliometric analysis utilized the Web of Science Core Collection (WoSCC) and Scopus as the primary data sources, both of which index high-quality, multidisciplinary journals and provide comprehensive bibliometric indicators. These indicators include details such as journal names, authors’ affiliations, countries/regions, publication years, keywords, and particular complete references. To address clinical research trends and improve methodological rigor, PubMed searches were used to supplement the data. Although PubMed lacks citation information, it remains prominent in medical bibliometrics due to three key advantages: its specialization in biomedicine; precise medical subject headings (MeSH) search functionality; and an open data policy.

Bibliometric methods offer the advantage of generating visual maps and cluster diagrams that highlight research themes and trends. However, since no universally adopted reporting guideline exists specifically for bibliometric reviews, we adhered to the PRISMA-ScR (Preferred Reporting Items for Systematic Reviews and Meta-Analyses extension for Scoping Reviews) statement as the most suitable framework to ensure transparent and comprehensive reporting (Tricco et al., 2018). To ensure the quality of our search and exclude irrelevant literature, we employed a combination of MeSH terms and free-text terms. The search strategy was as follow: (((“carbapenem resistan*” OR “imipenem resistan*” OR “meropenem resistan*” OR “ertapenem resistan*” OR “doripenem resistan*”) OR “carbapenemase-producing”) AND (“Gram-negative bacilli” OR “*Enterobacteriaceae*” OR “*K. pneumoniae*” OR “*A. baumannii*” OR “*Escherichia coli*” OR “*Klebsiella pneumoniae*” OR “*Salmonella*” OR “*Shigella*” OR “*Proteus*” OR “*Serratia*” OR “*Citrobacter*” OR “*Enterobacter*” OR “*Acinetobacter baumannii*” OR “*Pseudomonas*”)).

**Figure 1:**
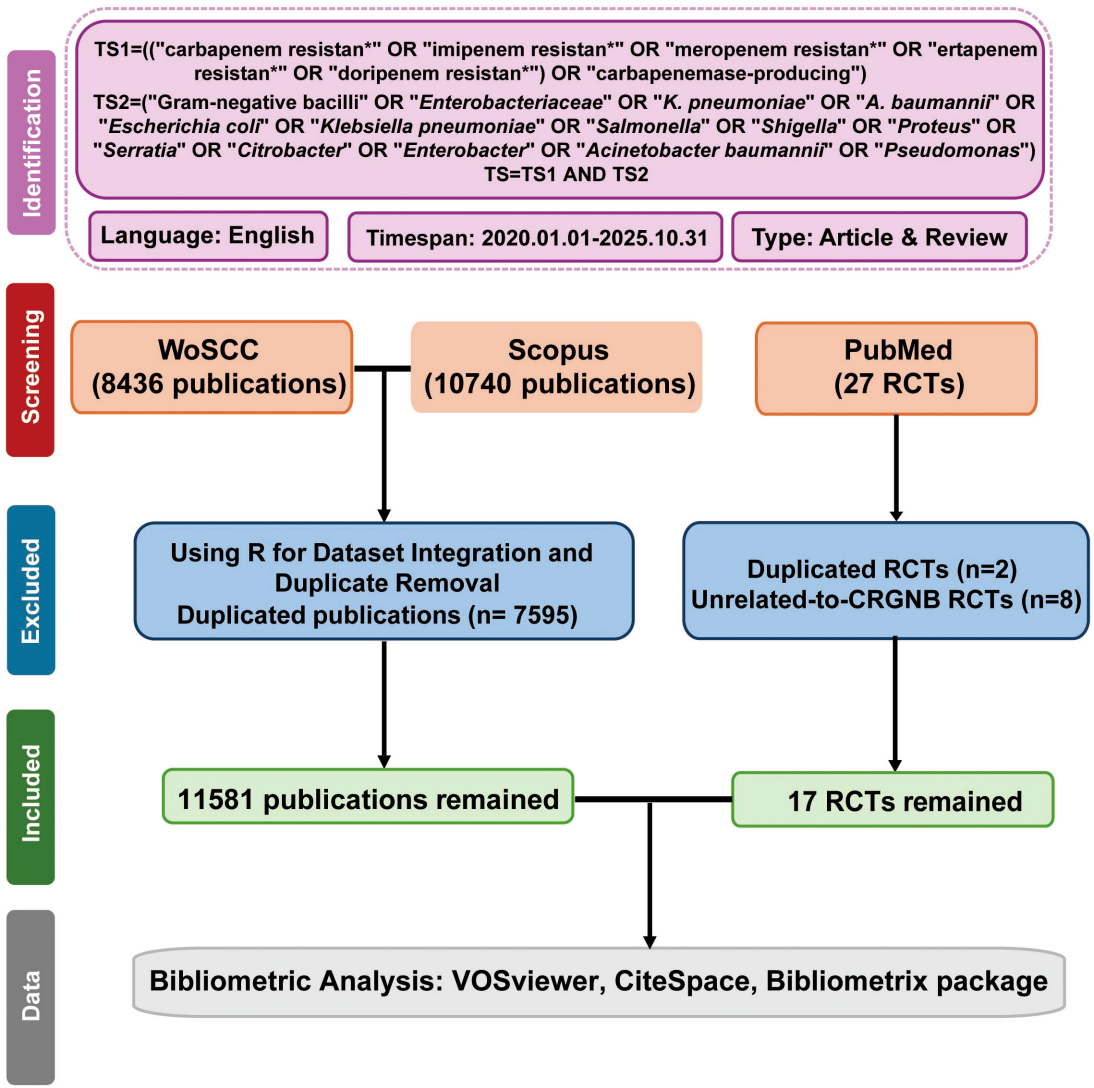
Flowchart of literature search and selection for CRGNB research. Literature was retrieved from WoSCC, Scopus, and PubMed, followed by dataset integration, duplicate removal, and screening of RCTs to obtain eligible publications for subsequent bibliometric analysis.

The search covered publications from January 1, 2020, to October 31, 2025, with the most recent update on November 1, 2025. Documents from the WoSCC were exported in “plain text” format, containing “full records and citations”. The documents exported from Scopus were in “CSV” and “BibTeX” formats. After exporting documents from WoSCC and Scopus in these formats, critical data extracted included: countries or regions, organizations, journals, authors, and keywords. The detailed search and analysis processes are depicted in the flowchart in **Figure 1**. To ensure rigorous quality control, two researchers independently reviewed and cross-verified all retrieved literature. If discrepancies arose regarding inclusion criteria, a third senior researcher was consulted to resolve any differences and reach a final consensus.

### 2.2 Literature cleaning and data processing

This study analyzed articles and reviews in the CRGNB field from the WoSCC and Scopus, while PubMed searches were specifically conducted for randomized controlled trials (RCTs). The search was limited to publications in English, and the following document types were excluded: (1) conference abstracts, news reports, letters, book chapters, and patents; (2) duplicate publications; and (3) documents unrelated to the research topic.

After data retrieval, the records were imported into CiteSpace (version 6.4.R1, developed by Drexel University, PA, USA) for deduplication and format conversion. The datasets were then combined and underwent an additional round of duplicate removal using R software. Subsequently, statistical compilation on countries, authors, keywords, and other relevant aspects was performed. To ensure consistency across the dataset, identical author and institution names were standardized by merging duplicate entries. Keywords were also filtered by consolidating singular and plural forms as well as synonyms to eliminate any bias in the analysis. To minimize the risk of bias caused by database update, all searches and literature screenings were completed on the same day.

### 2.3 Data analysis

In this study, several analytical and visualization tools were used, including VOSviewer (version 1.6.20, developed by the Centre for Science and Technology Studies at Leiden University, The Netherlands), CiteSpace, and the Bibliometrix package (based on R version 4.5.1 Bibliometrics package). These tools were employed for statistical analysis and bibliometric visualization.

VOSviewer is a specialized software for constructing and visualizing bibliometric networks. Thematic analysis was conducted by examining the co-occurrence of research topics to identify thematic similarities (van Eck and Waltman, 2010). In network visualization, each element—such as authors, institutions, or countries—is represented as a node, with the size of each node proportional to its frequency of their occurrence. The connections between nodes represent collaborative networks among these elements. Collaborative networks representing countries or regions were generated using VOSviewer, followed by the integration of these networks with the Charticulator tool to create chord diagrams illustrating national cooperation. Network visualization and overlay visualization of institutions were also created to explore institutional cooperation and publication timing. Additionally, a density visualization of journals was created to demonstrate the degree of co-citation among them.

CiteSpace software was used to plot collaboration networks, keyword clusters, and burst detection (Chen and Song, 2019). It is frequently employed to explore development trends and research progress in a specific field. CiteSpace was utilized to construct a collaborative network among authors, with particular attention to nodes exhibiting high centrality, which indicates their importance or influence within the network. Furthermore, a keyword cluster map was generated based on the log-likelihood ratio (LLR) to facilitate a more in-depth exploration of keywords.

The Bibliometrix package in R and its web interface “biblioshiny” were employed to analyze citation patterns, identify documents with high citation counts, and examine keyword trends in the literature. The Bibliometrix package was utilized to obtain the number of publications and citations for authors, institutions, countries or regions, and journals, and to calculate the h-index. Additionally, the package was applied to analyze the study’s themes.

## 3 Results

### 3.1 Global trend in publications outputs

**Figure 2:**
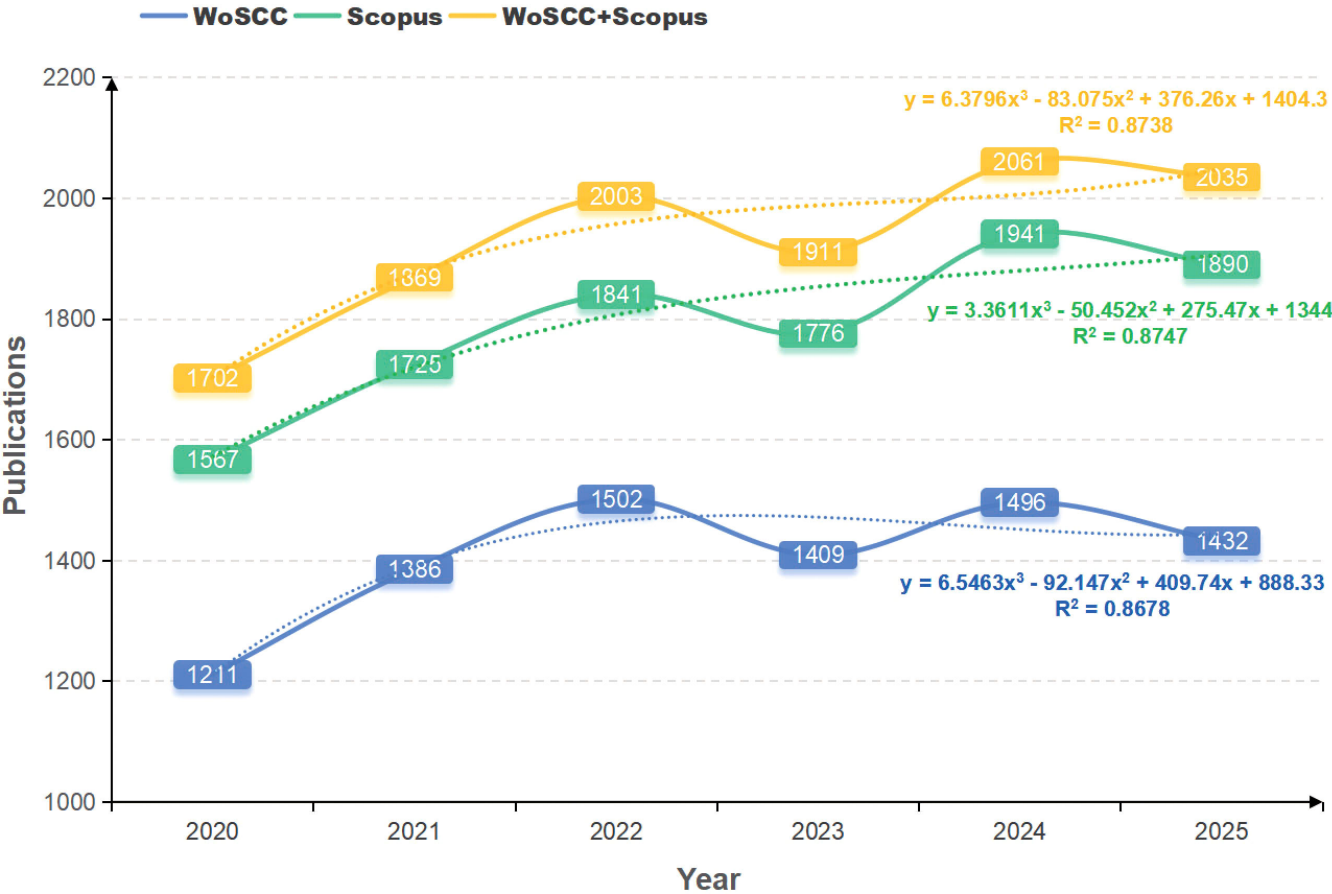
Annual publication trends of CRGNB research across different databases. Data cover the period from January 1, 2020, to October 31, 2025 (full-year 2025 data not yet available). Curves represent the fitted growth trends of publications in individual and combined databases, with corresponding R² values indicating goodness of fit.

The study comprised a total of 11,581 documents, including 10,568 articles and 1,013 reviews. The documents under scrutiny were authored by 40,145 individuals affiliated with 7,591 institutions across 124 countries or regions, and published in 1,486 journals. Changes in the number of publications within a given field over a specified period can serve as a reflection of the developmental status and trends within that particular field (Tomaszewski, 2023). The trends and statistics of research publications across different databases are illustrated in **Figure 2** and **Table 1**. To validate the integrated dataset, we compared the annual publication volume from WoSCC and Scopus. The results demonstrated highly consistent temporal trends between the three datasets, and the merged data exhibited a strong alignment with the projected growth curve. Over the past five years, the annual number of publications has shown an overall increasing trend despite fluctuation, suggesting a continuing rise in research activity in CRGNB. In 2024, the number of relevant literature publications reached a peak of 2,061, highlighting a significant surge in CRGNB-related research and the growing attention given to the issue of bacterial resistance. Although the 2025 data only cover the first ten months, the publication count is projected to approach or surpass the 2024 total, indicating that research output in the CRGNB field is likely to sustain this high level of activity.

**Table 1:** Overview of CRGNB researches in different databases.

Database	WoSCC	Scopus	WoSCC + scopus
Timespan	2020:2025	2020:2025	2020:2025
Documents	8,436	10,740	11,581
Articles	7,717	9,923	10,568
Reviews	719	817	1,013
Sources	847	1,381	1,486
Author’s Keywords	10,350	12,335	13,470
Single-authored documents	58	119	125
Co-Authors per documents	8.38	8.62	8.27
Annual Growth Rate (%)	3.35	3.82	3.59
Average citations per document	15.16	13.91	13.48

### 3.2 Analysis of authors

**Figure 3:**
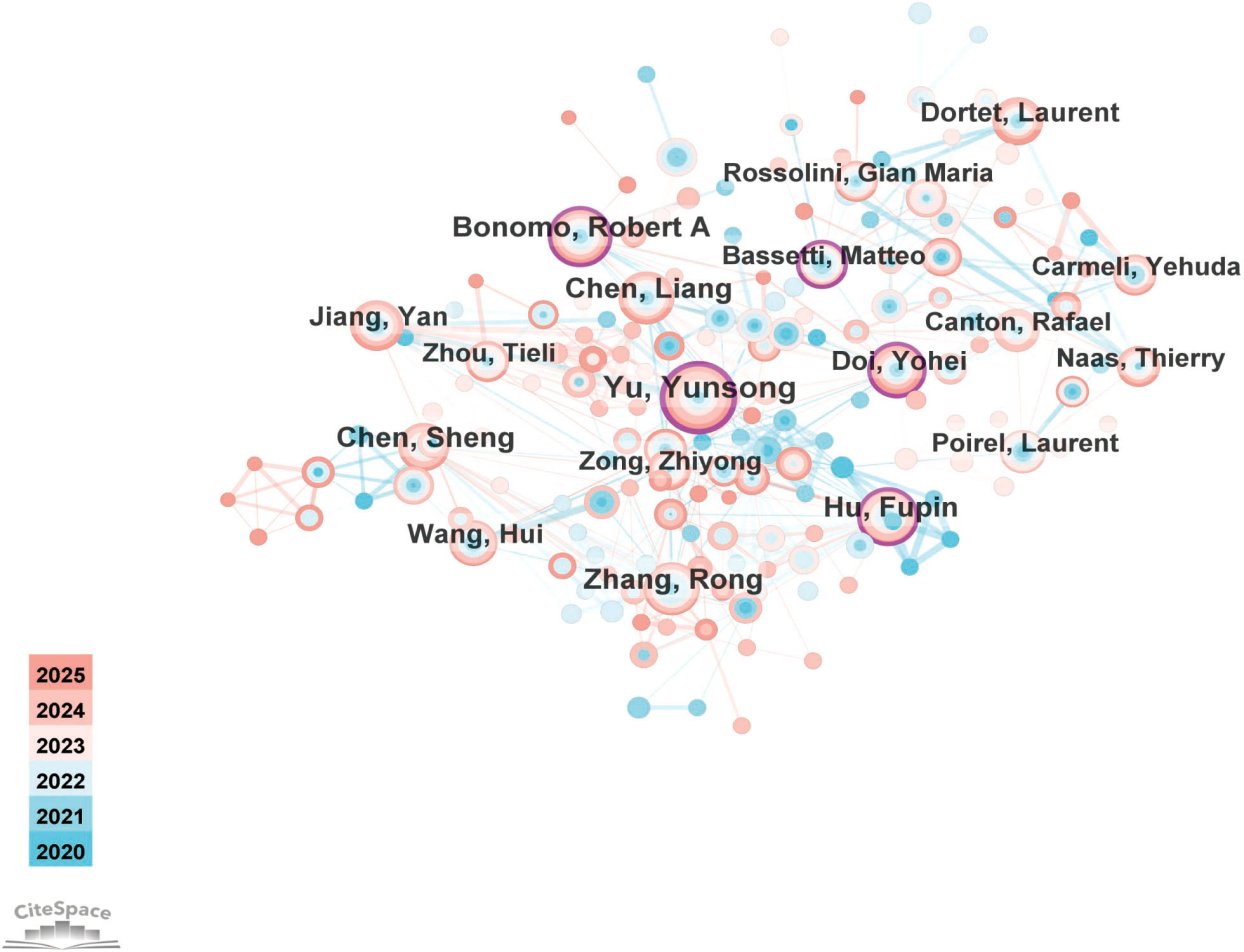
Author collaboration network in CRGNB research. Nodes represent authors, with node size proportional to the number of publications. Connection lines between nodes indicate collaborative relationships, and line thickness reflects the intensity of cooperation (frequency of co-authorship).

**Figure 3** illustrates the use of CiteSpace software to generate a visual representation of the author collaboration network, highlighting the cooperative relationships among authors within the field of CRGNB. The size of an author’s node is positively correlated with their number of publications, while the thickness of the connecting lines reflects the strength of collaboration between authors, measured by the frequency of co-authored publications. The total number of publications for each author is shown in **Table 2**.

**Table 2:** Academic contributions of the top 15 authors in CRGNB research.

Rank	Author	Publications	Citations	Average citations	h-index	Centrality
1	Yu, Yunsong	88	2,005	22.78	21	0.36
2	Zhang, Rong	72	2,139	29.71	20	0.08
3	Chen, Liang	67	1,294	19.31	22	0.04
4	Bonomo, Robert A.	62	3,598	58.03	23	0.11
5	Chen, Sheng	62	1,365	22.02	18	0.04
6	Hu, Fupin	62	1,565	25.24	17	0.18
7	Jiang, Yan	60	906	15.10	16	0.07
8	Castanheira, Mariana	54	1,042	19.30	19	0.01
9	Dortet, Laurent	53	773	14.58	15	0.01
10	Wang, Hui	53	1,172	22.11	20	0.06
11	Doi, Yohei	51	1,702	33.37	18	0.11
12	Naas, Thierry	47	1,372	29.19	17	0.06
13	Poirel, Laurent	47	753	16.02	15	0.06
14	Zhou, Tieli	47	538	11.45	14	0.02
15	Rossolini, Gian Maria	45	1214	26.98	23	0.04

Centrality is a metric that quantifies the frequency with which a node appears in the shortest path between any two nodes. The higher the centrality value, the more important the node is as a bridge in the network, indicating stronger internal connectivity. Yunsong Yu, positioned at the center of the network, maintains numerous cooperative relationships. With a centrality score of 0.36, he is closely connected to other authors. Yunsong Yu serves as a key figure in the research landscape of CRGNB. His work focuses on the resistance mechanisms and epidemiology of CRGNB, particularly CRKP. He has proposed strategies for rapid diagnosis and targeted treatment based on resistance and epidemiological characteristics, addressing the challenges posed by this resistance issue. The article authored by his team on hypervirulent CRKP has been extensively cited (Lan et al., 2021; Jin et al., 2021).

Similarly, Robert A. Bonomo occupies a significant position in the author collaboration network, with a centrality score of 0.11; he serves as a crucial bridge in collaborations with other authors. He has the highest number of citations, with an average citation count of 58.03, reflecting the widespread impact and reference value of his work. His research encompasses the molecular mechanisms of β-lactam antimicrobials and the molecular epidemiology of multi-drug resistant (MDR) Gram-negative bacteria (Tamma et al., 2024). Other authors, such as Doi Yohei, Fupin Hu and Matteo Bassetti have also garnered significant attention due to their extensive collaborative efforts.

The h-index is a metric that takes into account both the number of papers published by an author and the number of citations received by these papers. A higher h-index indicates greater academic influence in this field. Gian Maria Rossolini, Robert A. Bonomo, and Liang Chen have the highest h-index, with their research being highly relevant and timely.

### 3.3 Analysis of countries or regions

**Figure 4:**
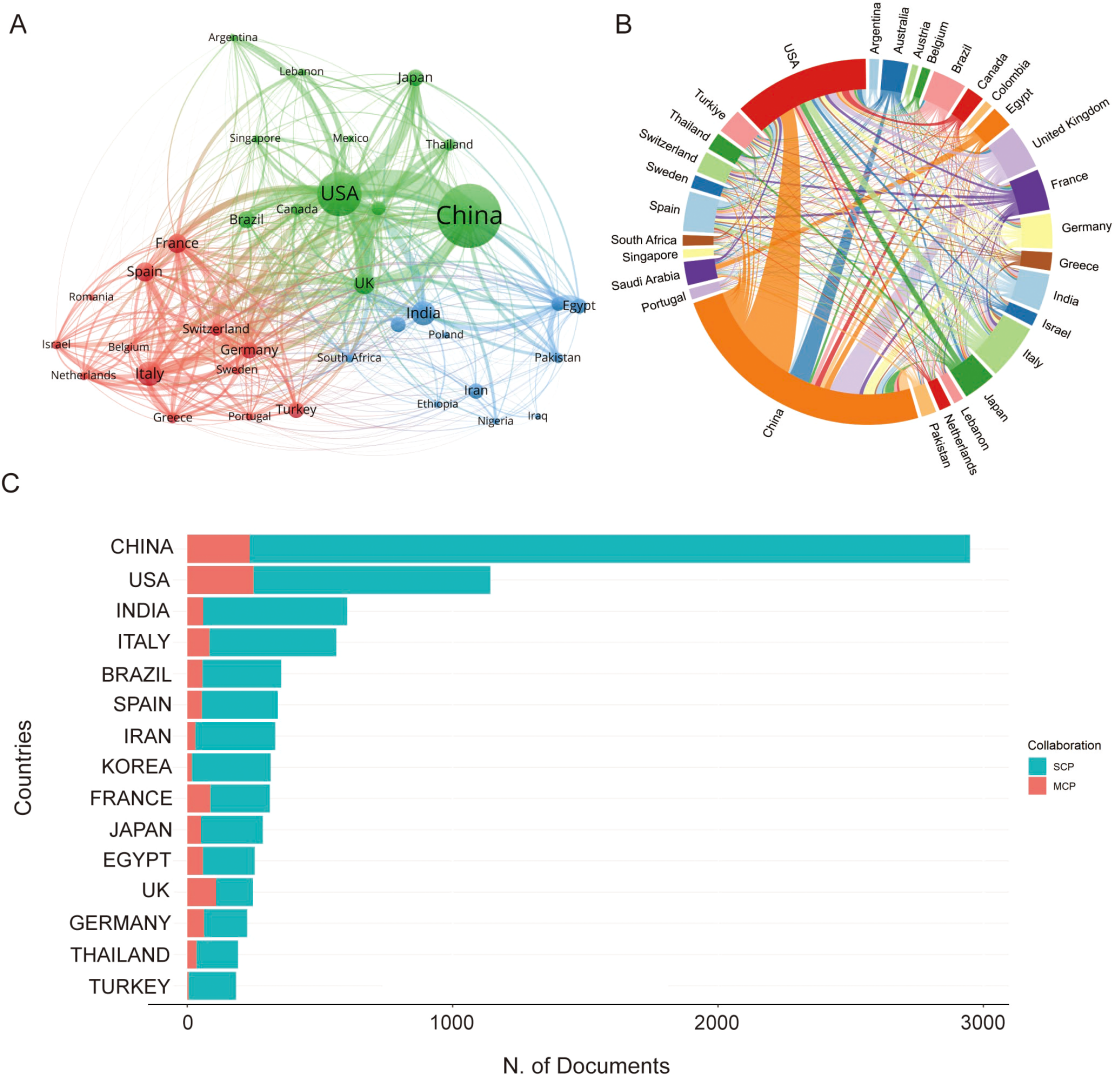
Publication output and international collaboration of countries/regions in CRGNB research. **(A)** Collaboration network map of the top 30 countries/regions with the highest publication volume. **(B)** Chord diagram illustrating the intensity of collaborative relationships among major countries/regions. **(C)** Distribution of SCP and MCP among the top 15 countries/regions, with MCP ratio reflecting the proportion of internationally collaborative publications.

This field has garnered widespread attention from the international community; however, there are noticeable disparities in the number of academic papers published across different countries and regions. As shown in **Figures 4A, B**, and **Table 3**, the five countries or regions with the highest number of publications are China (2,950 publications), the USA (1,143 publications), India (603 publications), Italy (562 publications), and Brazil (354 publications).

**Table 3:** Contribution of the top 10 countries or regions in CRGNB researches.

Countries or regions	Publications	Percentage of publications (%)	Single countries publication	Multiple countries publication	Multiple countries publication ratio
China	2,950	25.5	2,715	235	8
USA	1,143	9.9	894	249	21.8
India	603	5.2	543	60	10
Italy	562	4.9	479	83	14.8
Brazil	354	3.1	297	57	16.1
Spain	341	2.9	286	55	16.1
Iran	332	2.9	302	30	9
Korea	315	2.7	298	17	5.4
France	312	2.7	226	86	27.6
Japan	285	2.5	233	52	18.2

The network visualization map shows that the size of each node reflects the volume of publications, the thickness of the lines indicates the frequency of collaboration, and the color coding differentiates research clusters. In general, the majority of publications are concentrated in a few leading countries. China, with the highest number of publications, demonstrates the strongest collaboration network, particularly with the USA and the UK. It is also evident that countries such as Italy, France, Germany, and Spain, which are situated in the red cluster, maintain strong cooperative relationships among themselves. This suggests that a global collaborative network has been established.

**Figure 5:**
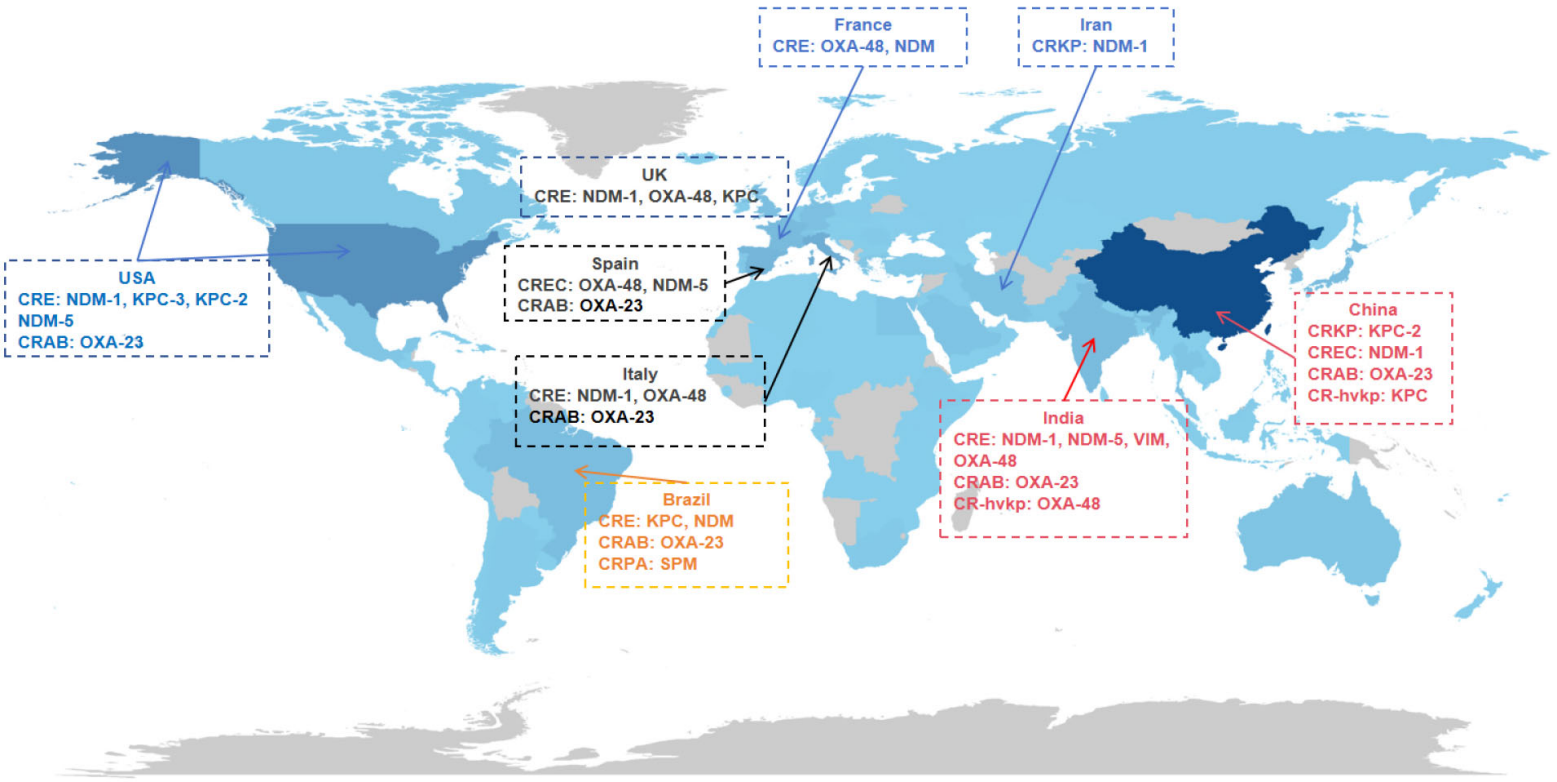
Summary of epidemiological characteristics of carbapenemase types in countries with high publication output on CRGNB. Notes: Color shade intensity corresponds to the publication volume of each country—higher shade intensity indicates a greater number of CRGNB-related publications from that country. KPC, *Klebsiella pneumoniae* carbapenemase; VIM, verona integron-encoded metallo-β-lactamase; NDM, New Delhi metallo-β-lactamase; OXA, oxacillinase; SPM, Sao Paulo metallo-β-lactamase.

**Figure 4C** illustrates the proportion of single-country publications (SCP) versus multiple-country publications (MCP). The top three countries in terms of the ratio of multilingual publications to total publications (MCP ratio) are France (27.6%), the USA (21.8%), and Japan (18.2%). Although China has published the highest number of papers, its MCP ratio remains relatively low, indicating that Chinese researchers still need to strengthen international cooperation. By adopting more innovative approaches and making breakthrough discoveries, Chinese researchers have the potential to enhance the global impact of their research in this field. To further clarify the global landscape of AMR, **Figure 5** presents a summary of the epidemiological characteristics of carbapenemase types in countries with high research output in the field of CRGNB. This visualization not only reflects the distribution differences of major carbapenemase subtypes across key research regions but also provides foundational data support for formulating region-specific AMR prevention and control strategies.

### 3.4 Analysis of institutions and journals

**Figure 6:**
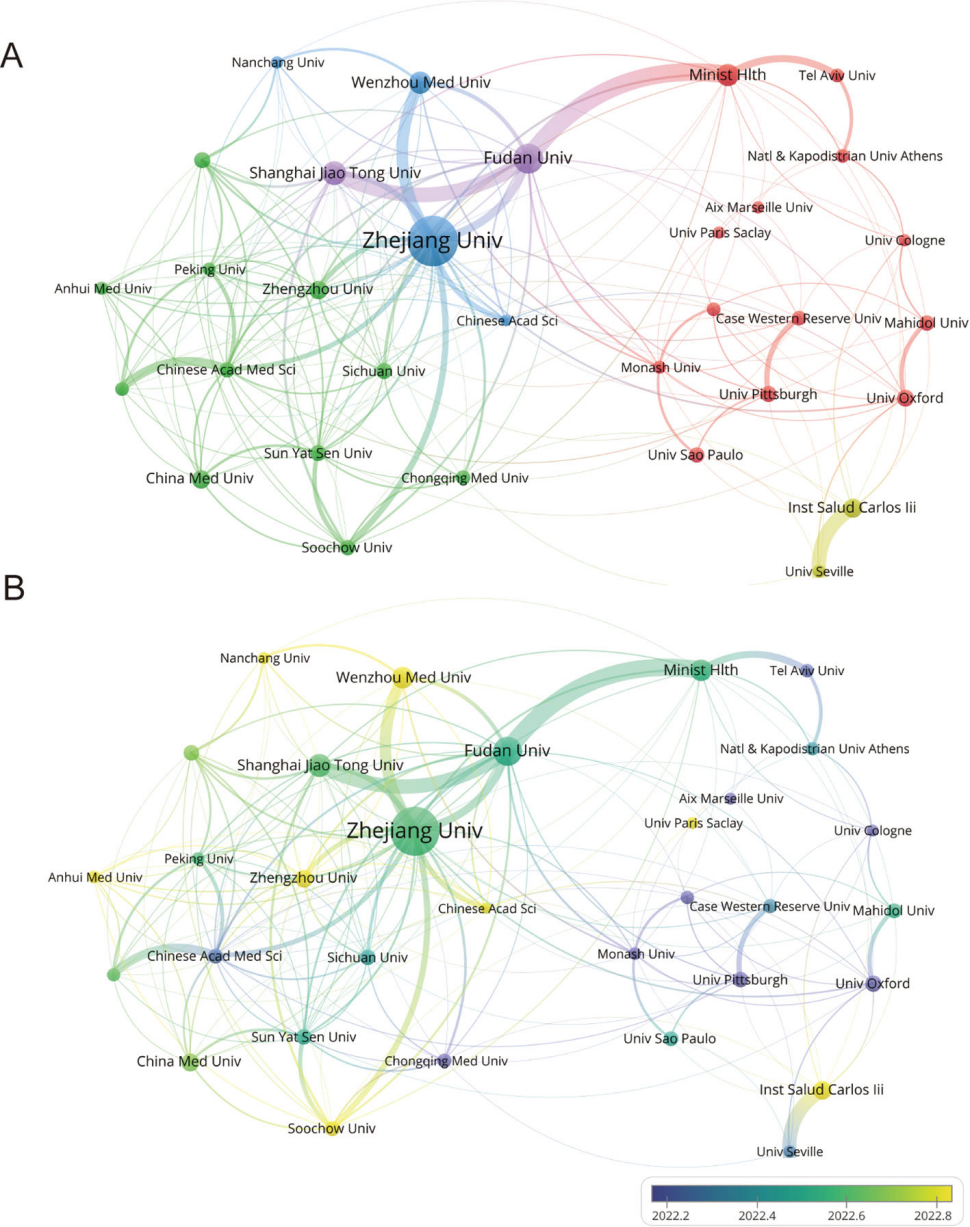
Institutional collaboration in CRGNB research. **(A)** Collaboration network among the top 30 institutions with the highest publication volume. Nodes represent institutions, and connection lines indicate collaborative relationships. **(B)** Overlay visualization of the top 30 institutions, showing the temporal distribution of their publication output.

The publication output of 7,591 institutions was analyzed in an effort to ascertain the major contributors to CRGNB research. Using VOSviewer software, we generated an institutional collaboration network (**Figure 6A**) and a time-evolution diagram (**Figure 6B**). The three institutions with the highest number of publications were Zhejiang University, Fudan University, and Shanghai Jiao Tong University, all of which are located in China. Regarding clustering and collaborative relationships, Zhejiang University is positioned within the blue cluster and maintains close collaborations with Wenzhou Medical University, the Chinese Academy of Sciences, and other institutions. This forms a broad cooperative network centered around Zhejiang University. Fudan University and Shanghai Jiao Tong University, placed in the purple cluster, collaborate with entities such as the Ministry of Health, Tel Aviv University and others. The time-evolution diagram highlights a significant concentration of publications from these institutions around the year 2022.

**Figure 7:**
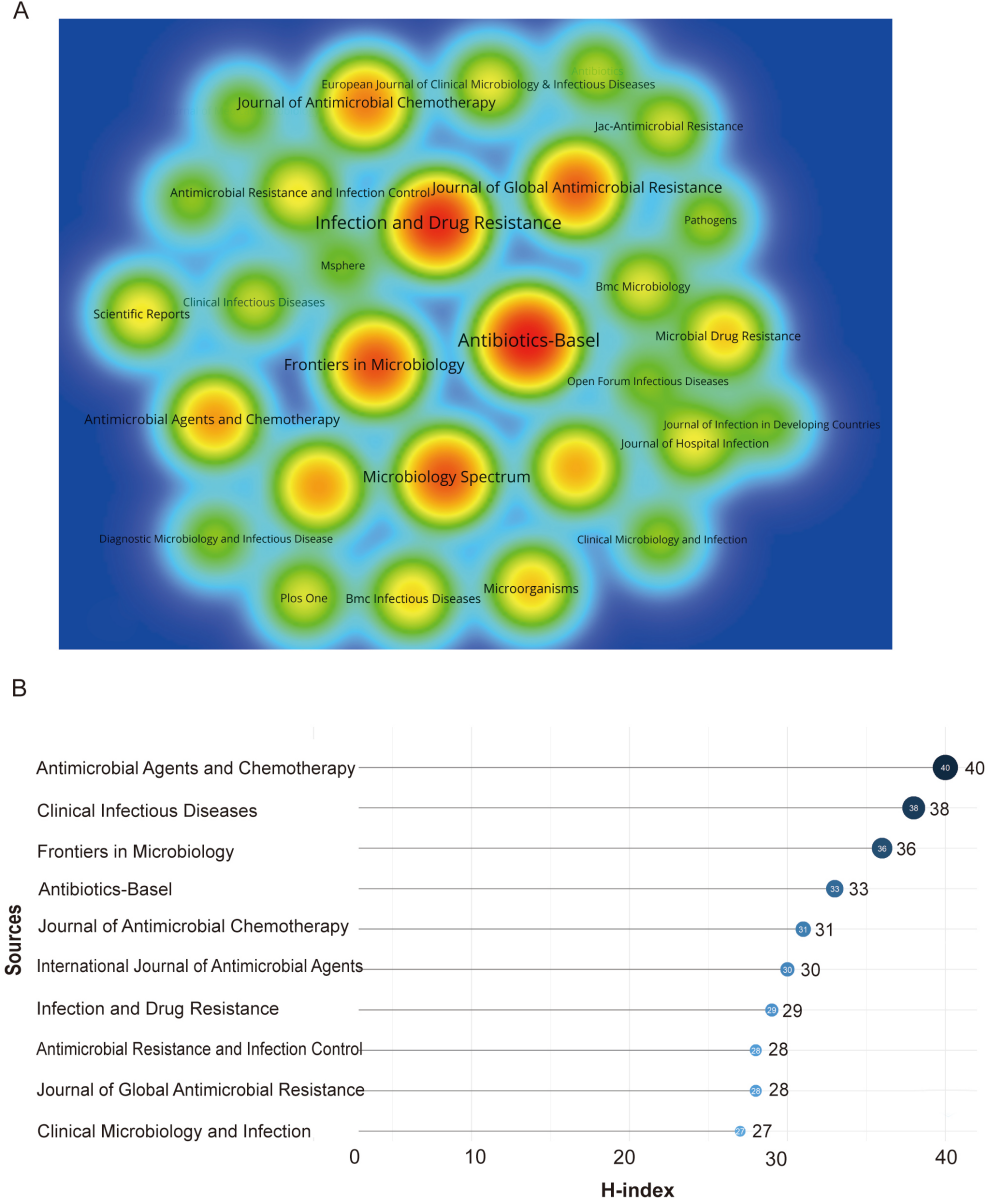
Journal-related visualization analysis of CRGNB research. **(A)** Density visualization of the top 30 journals publishing CRGNB-related literature, with color depth indicating the degree of co-citation among journals. **(B)** Ranking of the top 10 journals based on h-index, reflecting the academic influence of journals in the field.

**Figure 7A** illustrates a clear clustering phenomenon among the journals in which the literature was published. Additionally, **Figure 7B** shows the journal rankings based on the h-index, and **Table 4** provides a synopsis of the prevailing status of these journals. Notably, *Antibiotics (Basel)* and *Infection and Drug Resistance* published the highest volume of articles (577 and 516 publications, respectively), as evidenced by the darker hues in the spectrum. Furthermore, *Antibiotics (Basel)* has the highest number of citations, indicating that publications in this journal have a greater impact. Despite the relatively limited number of publications in *Antimicrobial Agents and Chemotherapy*, it has the highest h-index and a high number of citations, indicating high impact despite fewer publications, thus classifying it as a significant publication. Other journals, such as *Infection and Drug Resistance*, *Frontiers in Microbiology*, and *Clinical Infectious Diseases*, also exhibit higher citation rates, reflecting their authority and influence in the field of CRGNB research.

**Table 4:** Ranking of the top 10 journals in CRGNB research based on h-index.

Source	h-index	Publications	Citations	Journal citation reports	Impact factor
Antimicrobial Agents and Chemotherapy	40	246	5,429	Q1	4.5
Clinical Infectious Diseases	38	110	6,295	Q1	7.3
Frontiers in Microbiology	36	396	5,743	Q1	4.5
Antibiotics-Basel	33	577	7,782	Q1	4.6
Journal of Antimicrobial Chemotherapy	31	296	4,589	Q2	3.6
International Journal of Antimicrobial Agents	30	246	3,508	Q1	4.6
Infection and Drug Resistance	29	516	5,241	Q2	2.9
Antimicrobial Resistance and Infection Control	28	145	2,920	Q1	4.4
Journal of Global Antimicrobial Resistance	28	357	4,143	Q2	3.2
Clinical Microbiology and Infection	27	88	3,169	Q1	8.5

The data from the Journal Citation Reports and the Journal Impact Factor are derived from the 2024 edition.

### 3.5 Keyword analysis

**Figure 8:**
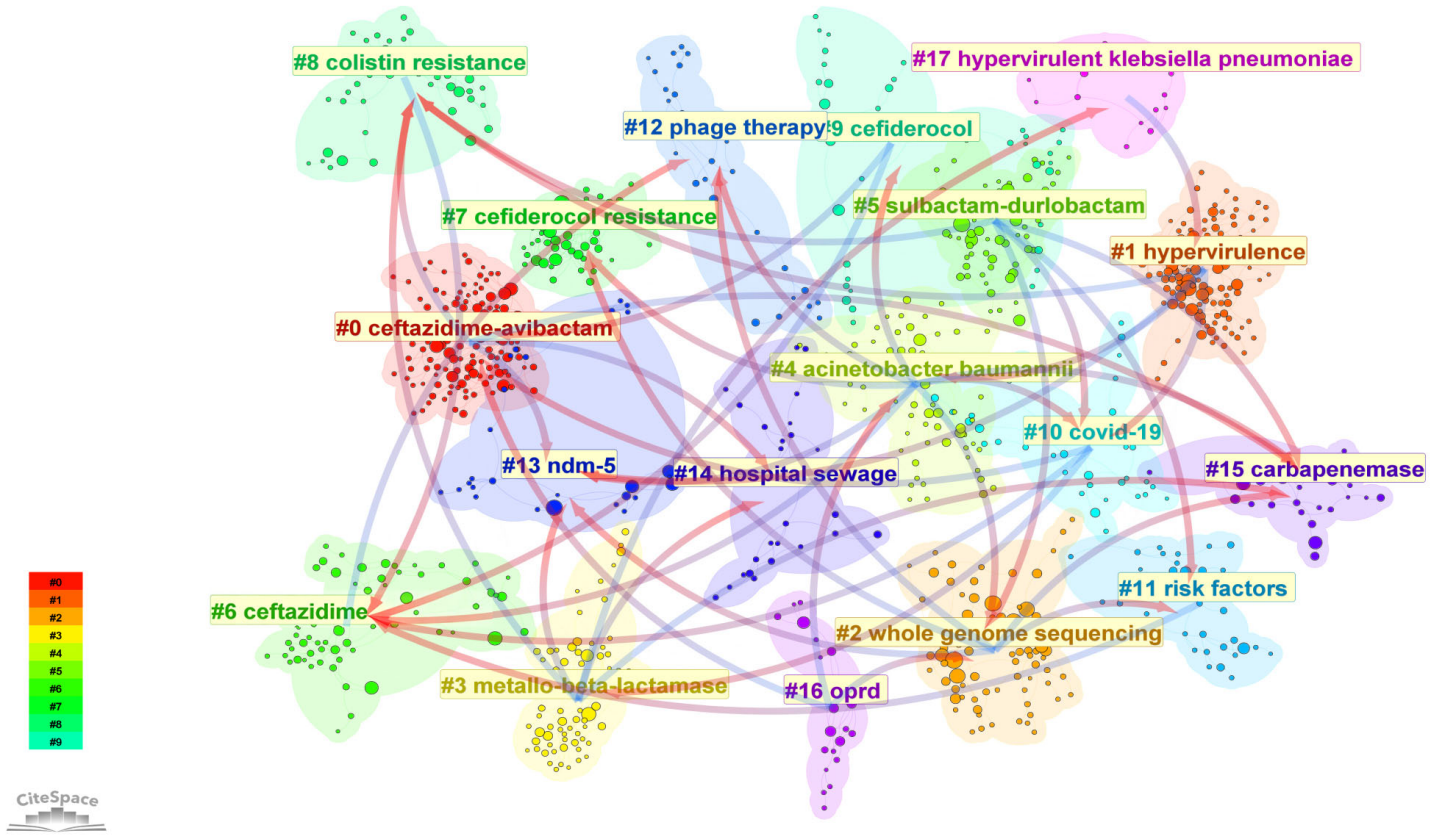
Thematic clusters of keywords in CRGNB research. Each cluster represents a core research topic, identified by unique colors. Keywords within the same cluster are thematically related, reflecting the main research directions in the field.

Using CiteSpace, the main keywords in CRGNB studies are displayed as clusters in **Figure 8**. Each cluster represents an independent research topic and is identified by a unique color for quick identification. Key clusters include: #1: hypervirulent CRKP and hypervirulent CREC, including cluster 1 (hypervirulence) and clusters 17 (hypervirulent klebsiella pneumoniae), highlighting on the emergence of hypervirulent strains of these bacteria. #2: resistance mechanisms, including cluster 15 (carbapenemase), cluster 13 (New Delhi metallo-β-lactamase [NDM]-5) and clusters 3 (metallo-β-lactamase [MBL]), emphasizing the various carbapenemases responsible for resistance in CRGNB. #3: antibiotic resistance, including cluster 7 (cefiderocol resistance) and clusters 8 (colistin resistance), addressing various forms of antibiotic resistance in CRGNB strains. #4: treatment strategies, including cluster 0 (ceftazidime-avibactam), clusters 9 (cefiderocol), clusters 5 (sulbactam-durlobactam) and clusters 12 (phage therapy), aiming to combat CRGNB infections. In addition to above four main clusters, cluster 16 (OprD [outer membrane protein D]) highlights the unique drug resistance mechanism of *Pseudomonas aeruginosa*, while cluster 14 (hospital sewage) underscores the “One Health” concept and its relation to environmental pollution issues associated with CRGNB. Both are also significant areas of concern.

### 3.6 Research hotspots and theme analysis

**Figure 9:**
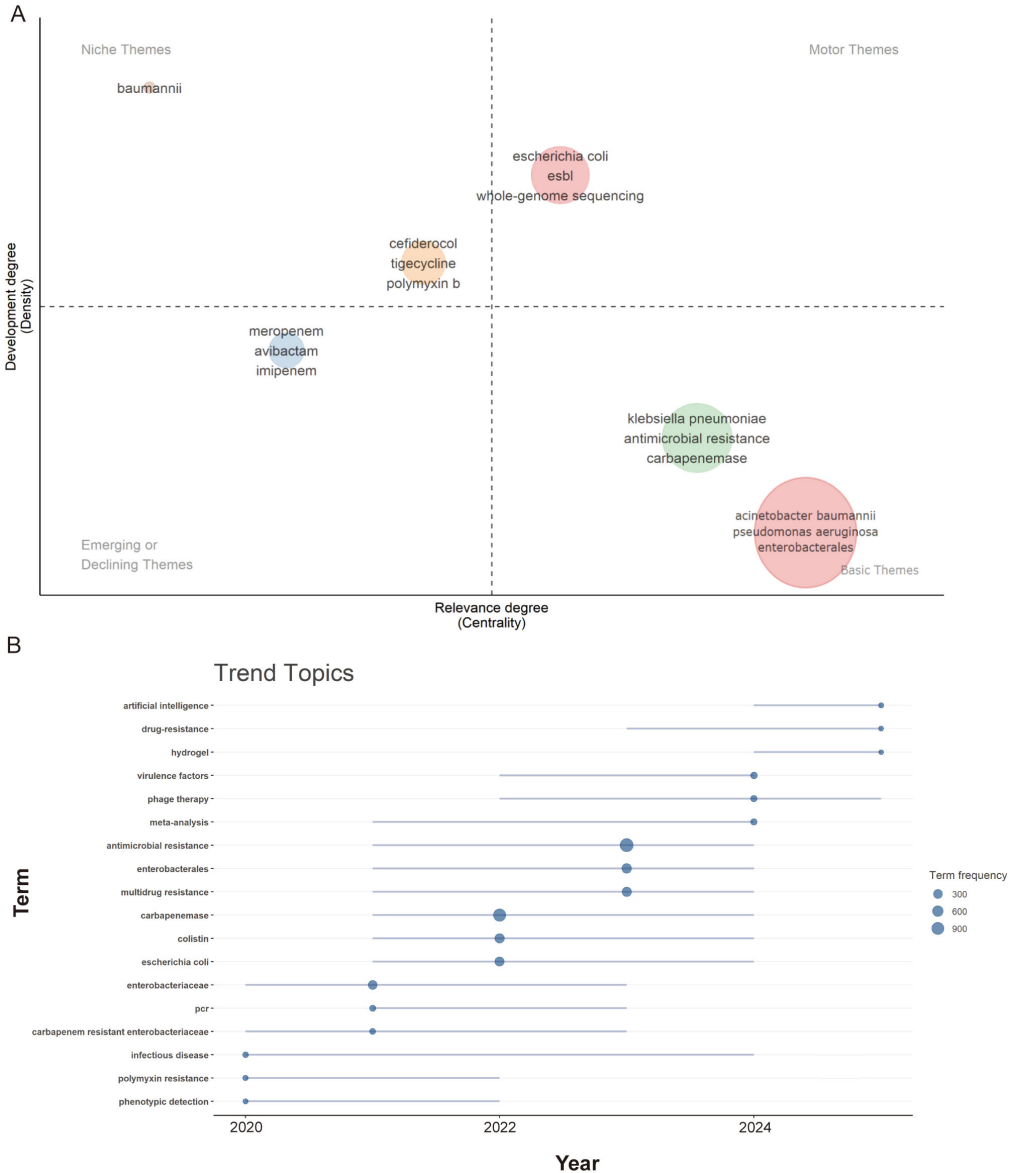
Analysis of research themes and trending topics in CRGNB research. **(A)** Thematic map based on author keywords, classifying research topics into four categories: Motor Themes (critical and rapidly developing), Basic Themes (foundational and well-established), Niche Themes (specialized and advanced), and Emerging or Declining Themes (peripheral or changing). The x-axis represents relevance degree (centrality) and the y-axis represents development degree (density). **(B)** Temporal evolution of trending topics based on keyword frequency, showing the shift in research focus from 2020 to 2025.

The thematic map, illustrated in **Figure 9A**, provides a visual representation of research hotspots. The study identifies four thematic trends: (1) Motor themes: These are crucial topics that show promising development trends. Keywords associated with motor themes include *E. coli*, extended-spectrum β-lactamase (ESBL), and whole-genome sequencing (WGS). This highlights the current focus on *E. coli* research, particularly on highly virulent carbapenem-resistant strains that have emerged in recent years. There is also a strong emphasis on developing methodologies for the early diagnosis and rapid screening of carbapenem resistance. Future research is expected to continue prioritizing this research area. (2) Basic themes: These are important but not yet fully developed areas of research. Research on *K. pneumoniae*, *A. baumannii*, *P. aeruginosa*, and carbapenemases remains foundational to CRGNB studies, with notable achievements in these areas. These studies constitute the bulk of the existing literature in the field. (3) Niche themes: These represent highly advanced yet isolated topics of research, such as cefiderocol, tigecycline, and polymyxin B. These domains have achieved significant progress but remain somewhat specialized. It is recommended that future research efforts focus on fostering collaboration among countries and institutions to consolidate knowledge on antimicrobial agents. (4) Emerging or declining themes: These themes represent weakly developed or peripheral topics that may either be emerging or in decline.

The changes in research topics, including shift in focus and emerging trends, were further analyzed (**Figure 9B**). From 2020 to 2023, the primary focus centered on core pathogens, drug resistance mechanisms, and detection methods, which form the foundational pillars of CRGNB research. Subsequently, research trends in 2024 are reflected in “phage therapy” and “meta-analysis”. Phage therapy, a promising alternative or adjunctive treatment strategy, has attracted growing interest and become a cutting-edge topic in the field. Additionally, meta-analysis has emerged as the dominant trend, reflecting the field’s maturity and the growing demand for high-quality, evidence-based summaries. Finally, the latest noteworthy topic is “artificial intelligence”, encompassing new predictive models and testing strategies that hold promise for alleviating the severe problem of drug resistance.

### 3.7 RCTs analysis

**Figure 10:**
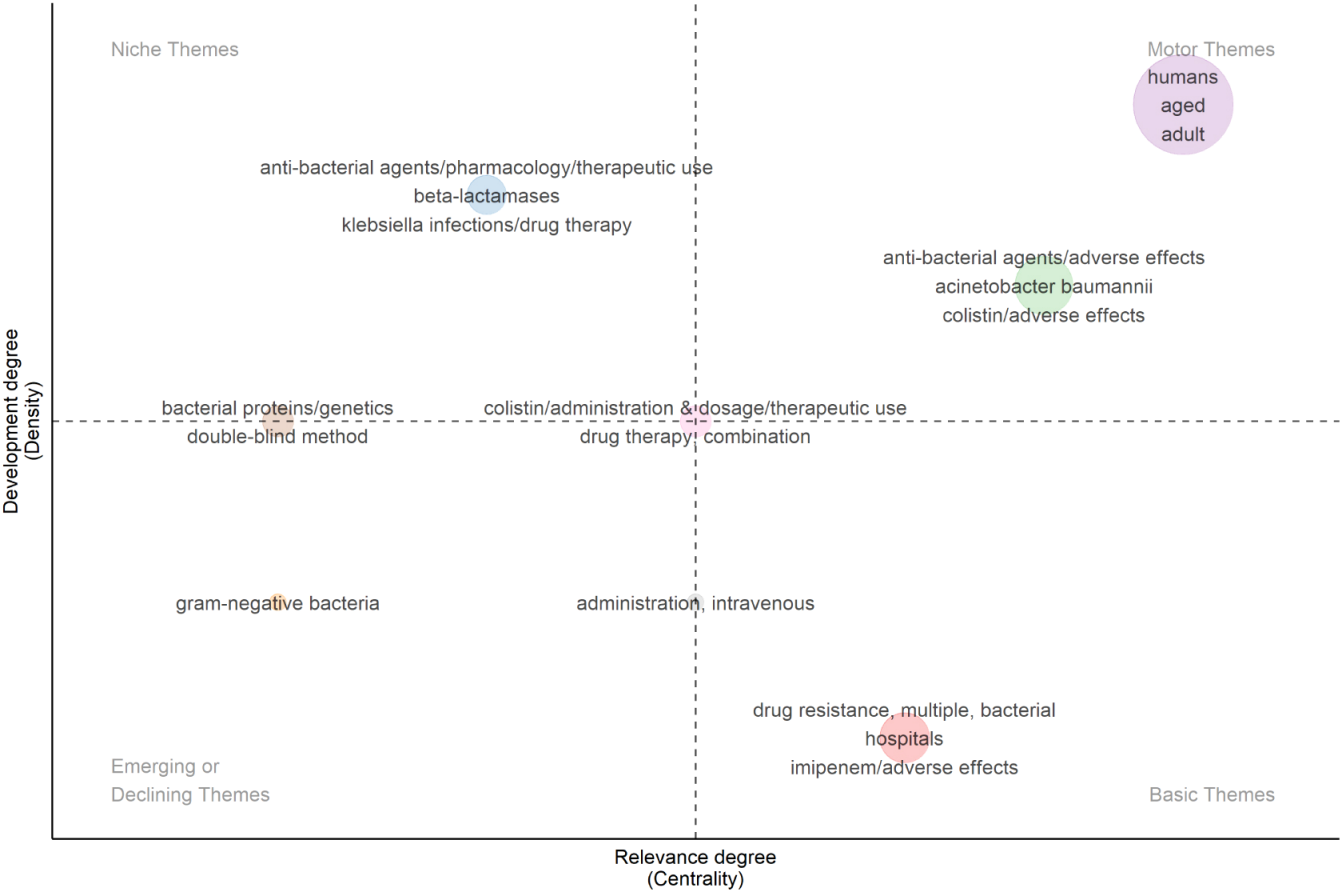
Thematic map of keywords from RCTs in CRGNB research. Keywords are clustered based on their relevance to RCTs, reflecting the core themes of clinical trial research, such as antimicrobial agents, treatment efficacy, and patient populations.

**Figure 11:**
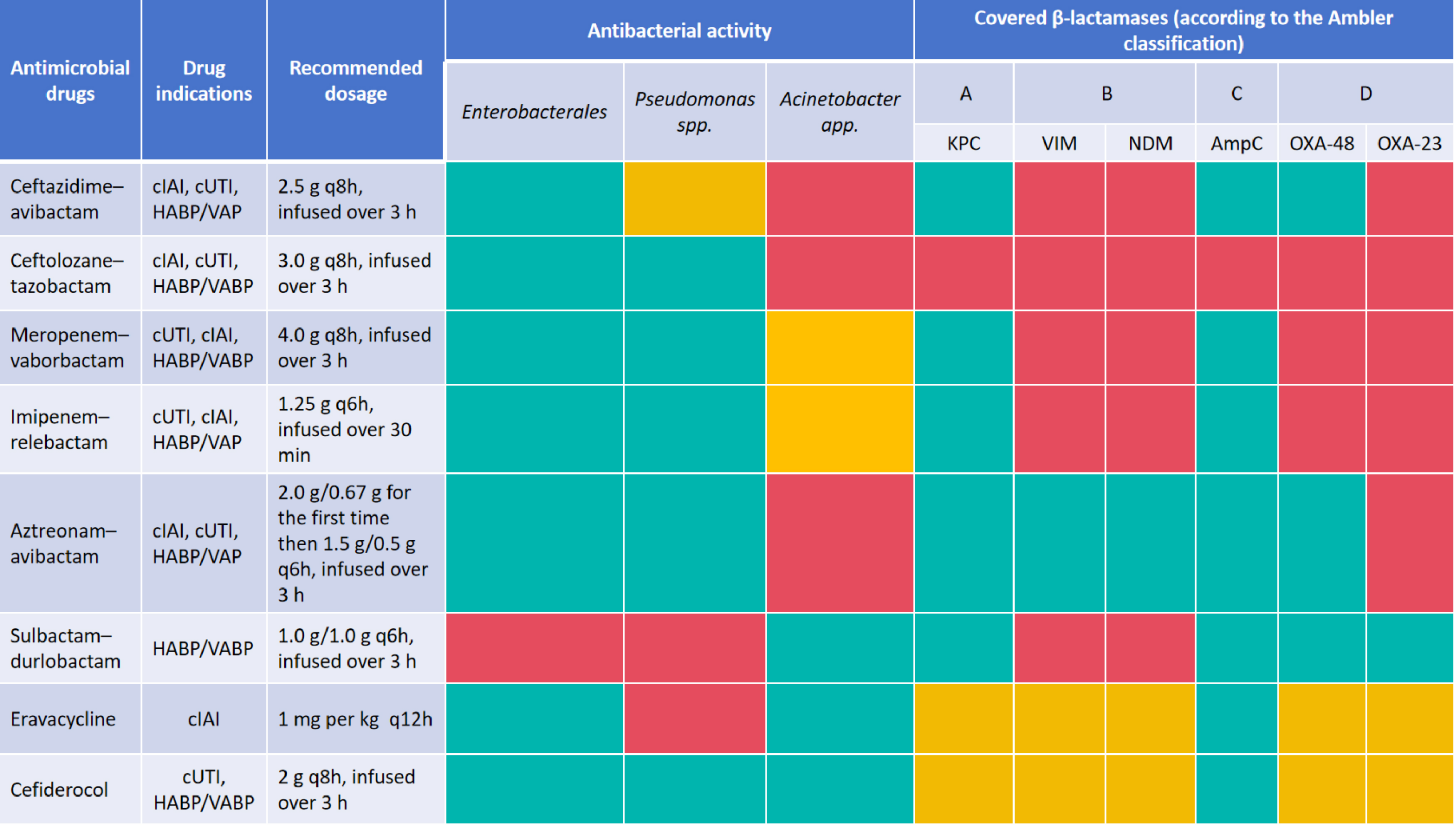
A summary of emerging and essential antimicrobial agents for CRGNB infections. “Green” indicates full antimicrobial drug coverage; “yellow” indicates partial antimicrobial drugs coverage; “red” indicates no antimicrobial drugs coverage; cIAI, complicated intra-abdominal infection; cUTI, complicated urinary tract infection; HABP, hospital-acquired bacterial pneumonia; VAP, ventilator-associated pneumonia; VABP, ventilator-associated bacterial pneumonia; KPC, *Klebsiella pneumoniae* carbapenemase; VIM, verona integron-encoded metallo-β-lactamase; NDM, New Delhi metallo-β-lactamase; AmpC, cephalosporinase; OXA-48, oxacillinase-48; OXA-23, oxacillinase-23.

A total of 27 RCTs were retrieved from the PubMed database. After excluding studies deemed irrelevant to the field, 17 RCTs were included in the analysis. The thematic maps in **Figure 10** highlight that the targeted development of antimicrobial drugs based on the mechanisms of bacterial resistance and genetic material is a potential direction for future drug research and development (motor themes). In addition, the central research themes in clinical trials related to CRGNB revolve around evaluating the efficacy and safety of antimicrobial agents targeting CRGNB infections (basic themes). The emerging and essential antimicrobial agents for CRGNB treatment are summarized in **Figure 11**. RCTs evaluating the efficacy and safety of new antimicrobial drugs and combination therapies, were major component, reflecting their increased clinical attention. This indicates that research on these new drugs will remain the primary focus moving forward.

We further present basic information on RCTs of new antimicrobial drugs (**Table 5**). The most-cited trial is a multicenter phase III clinical trial (Bassetti et al., 2021), which evaluated the efficacy of cefiderocol in treating CRGNB infections (Citations = 569). As the first iron-carrier cephalosporin, cefiderocol demonstrates significant antibacterial activity against CRGNB, particularly those producing MBLs. This offers a novel treatment option for infections caused by MDR bacteria. This trial involved 152 adult patients who were randomly assigned to either the cefiderocol monotherapy group or the best available therapy group. The results indicated that cefiderocol showed comparable clinical and microbiological efficacy to the best available therapy. However, although the overall mortality rate was higher in the cefiderocol group (34% vs. 18%), this difference was primarily observed in critically ill patients with *A. baumannii* infection. These safety concerns require further investigation.

**Table 5:** Summary of randomized controlled trials on novel antimicrobial agents for carbapenem-resistant Gram-negative bacteria infection.

References	Study name	Study design	Participants	Intervention	Comparison	Primary outcomes	Safety	Notes
Motsch et al., 2020	RESTORE-IMI 1	Randomized, double-blind, phase 3 trial	• Adults with imipenem-nonsusceptible HAP/VAP, cIAI, or cUTI	• Imipenem/relebactam 500/250 mg IV q6h for 5–21 days (for ≥ 5 days [cIAI, cUTI] or 7 days [HAP/VAP])	• Colistimethate sodium (loading dose 300mg CBA, then 150mg CBA) IV q12h + imipenem 500mg IV q6h	• Comparable clinical response (71% vs. 70%) • Lower mortality (10% vs. 30%) with imipenem/relebactam	• Fewer severe AEs (10% vs. 31%), drug-related AEs (16% vs. 31%), and nephrotoxicity (10% vs. 56%)	• Relebactam inhibits KPC and other β-lactamases (ESBLs and AmpC)
Bassetti et al., 2021	CREDIBLE-CR	Randomized, open-label, multicenter, pathogen-focused, phase 3 trial	• Hospitalized adults (≥18 years) with HAP, VAP, HCAP, BSI, sepsis, or cUTI caused by CRGNB	• Cefiderocol 2 g IV q8h (adjusted for renal function) for 7–14 days • Cefiderocol combined with one adjunctive antibiotic for HAP, BSI or sepsis, excluding polymyxins, cephalosporins, and carbapenems	• Best available therapy for 7–14 days	• Clinical and microbiological outcomes similar to best available therapy; higher all-cause mortality in cefiderocol group (34% vs. 18%), especially in CRAB infections	• Comparable TEAEs (91% vs. 96%)	• First iron-carrier cephalosporin • Activity against MBL producers
Titov et al., 2021	RESTORE-IMI 2	Randomized, controlled, double-blind, phase 3 trial	• Adults with HABP or VABP	• Imipenem/cilastatin/relebactam 500/500/250 mg IV q6h for 7–14 days	• Piperacillin/tazobactam 4/0.5 g IV q6h for 7–14 days	• Non-inferior 28-day all-cause mortality (15.9% vs. 21.3%)	• Comparable serious AE (26.7% vs. 32.0%), AEs-related treatment discontinuation (5.6% vs. 8.2%), and drug-related none fatal AEs (11.7% vs. 9.7%)	• Includes relebactam to overcome resistant strains • Gram-negative bacilli constituting 75.7% of pathogens, but with unreported proportion of carbapenem-resistant strains
Bruss et al., 2021	None	Phase 1, randomized, placebo-controlled, first-in-human trial	• Healthy adult subjects aged 18–55 years with a BMI of 18.5-29.9 kg/m² and weight between 55–100 kg	• SPR206 IV dosing regimens of single ascending doses (10–400 mg) and multiple ascending doses (25–150 mg q8 h for 7 days and 100 mg q8h for 14 days)	• Placebo (saline)	• Well-tolerated • No nephrotoxicity • Achieved target concentrations	• Mild, dose-dependent AEs	• Novel polymyxin derivative with improved safety profile
Kaye et al., 2023	ATTACK	Multicenter, randomized, active-controlled, non-inferiority, phase 3 trial	• Adults with HABP, VABP, VAP, or BSI caused by CRAB	• Sulbactam-durlobactam 1/1 g IV q6h + imipenem/cilastatin 1/1 g IV q6h for 7–14 days	• Colistin (loading dose 2.5-5g/kg, then 2.5mg/kg) IV q12h + imipenem-cilastatin 1g/1g IV q6h for 7–14 days	• Non-inferior 28-day all-cause mortality (19% vs. 32%) • Higher clinical (62% vs. 40%) and microbiological (68% vs. 42%) cure rates	• Lower serious AEs (40% vs. 49%), treatment-related AEs (11% vs. 16%), and nephrotoxicity (13% vs. 38%)	• Targeted activity against OXA-23-producing CRAB
Moeck et al., 2024	CERTAIN-1	Double-blind, randomized, phase 3 study	• Patients with cUTI (including acute pyelonephritis)	• Cefepime/taniborbactam 2/0.5 g IV q8h for 7–14 days	• Meropenem 1 g IV q8h for 7–14 days	• High composite (clinical and microbiological) success rates in CRE (87.5% [7/8]) and Enterobacterales with carbapenemase genes (OXA-48, KPC-3, NDM-1) (88.9% [8/9])	• Comparable AEs (35.5% vs. 29.0%)	• Taniborbactam broadens activity against serine β-lactamases

HAP, hospital-acquired pneumonia; VAP, ventilator-associated pneumonia; cIAI, complicated intra-abdominal infection; cUTI, complicated urinary tract infection; HCAP, healthcare-associated pneumonia; BSI, bloodstream infection; HABP, hospital-acquired bacterial pneumonia; VABP, ventilator-associated bacterial pneumonia; BMI, body mass index; CRAB, carbapenem-resistant *Acinetobacter baumannii*; CRE, carbapenem-resistant *Enterobacteriaceae*; OXA-48, oxacillinase-48; OXA-23, oxacillinase-23; KPC, *Klebsiella pneumoniae* carbapenemase; NDM-1, New Delhi metallo-β-lactamase-1; ESBL, extended-spectrum β-lactamase; AmpC, cephalosporinase; MBL, metallo-β-lactamase; IV, intravenous infusion; CBA, colistin base activity; AE, adverse event; TEAE, treatment-emergent adverse event; CRGNB, carbapenem-resistant Gram-negative bacteria; SPR206, a novel polymyxin derivative.

The second most-cited RCT was a phase III trial (Motsch et al., 2020) comparing the efficacy of the novel antibiotic combination imipenem-relebactam with the traditional therapy of colistin plus imipenem for treating CRGNB infections (Citations = 294). The findings showed that both groups exhibited comparable efficacy. However, the imipenem-relebactam group demonstrated significantly better outcomes in terms of reduced mortality and a lower risk of nephrotoxicity compared to the control group.

Next, a multicenter phase III non-inferiority clinical trial (Kaye et al., 2023) compared the efficacy and safety of sulbactam-durlobactam versus colistin in treating patients with serious infections caused by *Acinetobacter calcoaceticus-baumannii* (ACB) complex (Citations = 141). The findings demonstrated that sulbactam-durlobactam was non-inferior to colistin when both agents were administered in combination with imipenem-cilastatin, based on the primary endpoint of 28-day all-cause mortality.

## 4 Discussion

The global rise of CRGNB represents one of the most pressing challenges in modern infectious disease management. Mortality rates reach up to 50% in vulnerable populations, and the economic burdens on healthcare systems worldwide is substeantial (Madney et al., 2023). Our bibliometric analysis of 11,581 publications from January 1, 2020 to October 31, 2025, combined with a comprehensive synthesis of recent clinical evidence, reveals a continuous year-over-year increase in CRGNB research. This growth reflects the urgent need to address this escalating AMR crisis. In this discussion, we systematically analyze and summarize the key findings from our bibliometric analysis and current research progress, providing valuable insights into global research trends, epidemiology, resistance mechanisms, diagnostic approaches, therapeutic strategies, and future directions in CRGNB research.

### 4.1 Global research output and collaborative networks

The geographical distribution of CRGNB research reveals striking disparities in both productivity and impact, which mirror the epidemiological patterns of resistance. China’s remarkable output of 2,950 publications reflects its substantial investment in AMR research; however, its MCP ratio—indicating the proportion of internationally collaborative publications—lags behind that of the USA. This difference may reflect variations in research focus, with Chinese studies frequently emphasizing epidemiological surveillance, while the USA prioritizes mechanistic and therapeutic innovation. These research patterns align with the global resistance epidemiology described by Jean et al. (2022) and Nordmann et al (Nordmann and Poirel, 2019), in which *Klebsiella pneumoniae* carbapenemase (KPC)-producing strains prevail in China and Southern Europe, NDM-β-lactamase variants dominate South Asia and the Middle East, and oxacillinase (OXA)-48 producers are most common in Europe. A multicenter cohort study found significant global variability in CRKP infections, including higher 30-day mortality rates in South America, more severe comorbidities in USA patients, and higher detection rates of virulence genes in Chinese strains (Wang et al., 2022). International collaborations, exemplified by networks centered on researchers such as Yunsong Yu (China) and Robert A. Bonomo (USA) have been instrumental in advancing the field.

The emergence of specialized research hubs has been critical in driving progress in this area. Zhejiang University’s leadership in CRGNB research reflects China’s focused response to its domestic CRKP epidemic and fosters close collaboration with institutions such as Wenzhou Medical University and the Chinese Academy of Sciences. Moreover, the WHO’s 2024 priority pathogen list has further catalyzed these collaborations, particularly in addressing critical gaps in diagnostics and therapeutics for CRE, CRAB, and CRPA (Jesudason, 2024). Together, these centers have formed a robust cooperative network, centered on Zhejiang University, which not only produces a high volume of publications but also serves as an integral node in global collaboration networks. Such networks are essential for tackling a problem that transcends national borders.

The relatively low MCP ratio for China can be attributed to several interconnected factors. First, research agendas in China mainly prioritize domestic public health needs, driven by the country’s unique CRGNB epidemic landscape, such as the high prevalence of sequence type (ST) 11-KPC-2 CRKP strains (Qin et al., 2024). National surveillance networks like CHINET and key AMR funding initiatives focus on domestic clinical challenges, such as epidemiological monitoring and nosocomial transmission control, thereby limiting the availability of resources for international collaboration. Second, linguistic and cultural barriers, despite widespread English proficiency in top-tier institutions, hinder cross-border cooperation due to differences in academic norms and limited overseas collaboration networks. Third, the COVID-19 pandemic (2020–2023) not only disrupted cross-border academic exchanges but also resulted in national research funding to be redirected toward domestic public health prevention and control efforts, further reducing opportunities for international collaboration. Finally, national funding mechanisms prioritize domestic application value. As a result, only a limited number of international collaborative projects receive support, creating a structural disincentive for cross-border cooperation.

### 4.2 Resistance mechanisms and epidemiology

Resistance mechanisms remain a central focus of CRGNB research. In the case of CRE, significant attention has been paid to the rapid spread of CRKP and CREC strains producing carbapenemases such as KPC, NDM, and OXA-48 (Han et al., 2020). Globally, CRKP accounts for over 70% of CRE infections, with sequence types (ST) 258 and ST 11 emerging as the predominant clones (Balushi et al., 2022). The KPC-2 isoenzyme is the dominant variant worldwide, particularly in China, Southern Europe, and South America, where it accounts for more than 80% of CRKP isolates in China (Hu et al., 2024). In contrast, KPC-3 is more prevalent in North America and Western Europe, where it exhibits lower resistance to ceftazidime-avibactam (Chen et al., 2022). Notably, China has experienced a surge in *bla*_KPC_ variants since 2020. This increase followed the approval of ceftazidime-avibactam for clinical use in 2019 (Ding et al., 2023). While ceftazidime-avibactam remains effective against most KPC-producing strains (Boattini et al., 2023), resistance caused by porin loss or mutations reduces the efficacy of carbapenems in treating these infections (Shen et al., 2025). Although CREC remains less common, its prevalence is increasing in Asia, often linked to the ST410 clones (Ba et al., 2024). Studies by Shields et al. (2017) and Tang et al. (2024) have documented the remarkable adaptability of KPC enzymes, noting rapid mutations in the *bla_KPC_* gene under selective pressure from novel β-lactamase inhibitors.

Carbapenem-resistant and hypervirulent *K. pneumoniae* (CR-hvKP) represents a particularly formidable challenge in bacterial evolution, marked by the convergence of hypervirulence and carbapenem resistance. This phenomenon can arise through three main pathways: (1) hypervirulent *K. pneumoniae* (hvKP) strains with capsular serotypes K1/K2 acquiring carbapenem-resistant plasmids (known as CR-hvKP); (2) CRKP strains acquiring virulence plasmids (known as hv-CRKP); or (3) hvKP or CRKP strains obtaining a fusion plasmid that contains both carbapenem resistance genes and virulence genes (Wang et al., 2025b). This highlights the critical role that plasmids play in the dissemination of both drug resistance and virulence. In July 2024, the WHO reported a rising global incidence of ST23 and K1/K57 CR-hvKP strains (World Health Organization, 2024b). Recent studies by Pu et al. (2023) attribute this surge to mobile genetic elements, such as plasmids and transposons, which facilitate rapid gene transfer between bacterial species. China remains the country with the highest prevalence of CR-hvKP, where the primary transmission type in China is ST11-KL64 CR-hvKP, as confirmed by recent studies from Chen et al. (2025) and (Liu et al., 2025). The widespread dissemination of ST11 CR-hvKP strains in China is largely attributed to horizontal transmission mediated by incompatibility group F (IncF) plasmids, particularly among KPC-producing CRKP strains (Yang et al., 2022). A recent report by Jiang et al. (2025) indicated that an IncFIIK34 KPC-2 plasmid has a higher conjugation frequency and a competitive advantage compared to other plasmids, which could be a key factor in the global spread of CR-hvKP. Consequently, a bioconjugate vaccine targeting K1/K2 serotypes has been developed for hvKP infections (Feldman et al., 2019). Exploring the mechanisms behind the acquisition of both virulence and drug-resistance genes could provide innovative strategies for the development of novel therapeutic interventions.

Research on CRPA emphasizes the role of intrinsic resistance mechanisms, including efflux pump overexpression (e.g., MexAB-OprM) and porin mutations (e.g., OprD loss) (Muderris et al., 2018), as well as acquired carbapenemases (verona integron-encoded MBL [VIM], imipenemase [IMP], and KPC) (Karampatakis et al., 2025). A multinational study revealed carbapenemase genes in 22% of CRPA isolates, predominantly KPC-2 (49%) and VIM-2 (36%) among carbapenemase-positive isolates. Significant geographical variations were observed, with high prevalence in South and Central America (69%) and Australia and Singapore (57%), but low prevalence in the USA (2%) (Reyes et al., 2023). A recent study reported a clonal outbreak of ST313 *P. aeruginosa* strains co-producing IMP-45 and VIM-1 carbapenemases in China (Ma et al., 2025). The success of ceftolozane-tazobactam against many CRPA isolates has been countered by emerging resistance, particularly in strains with AmpC overexpression and MBLs production (González-Pinto et al., 2025). MBLs have attracted widespread attention due to their association with high mortality and increased intensive care unit admission rates, as demonstrated by numerous reports from regions across Europe, the Asia-Pacific, North America, Latin America, and Africa (Grabein et al., 2024). A recent study documented an outbreak of MBL-producing *P. aeruginosa* at a hospital in Southern California (Garrigues et al., 2025). Additionally, an epidemiological study analyzed 2,639 *bla_MBL_*-carrying *P. aeruginosa* strains, with the highest number of strains reported from the USA, Singapore, Germany, and Poland (Zhai et al., 2025).

Studies on CRAB underscore the global dominance of OXA-23, OXA-58, and NDM-1 carbapenemases, which are present in over 80% of clinical isolates collectively (Asif et al., 2018). A substantial international multicenter study has determined that the clonal group 2 (CG2) predominates on a global scale among CRAB, with the exception of South America (Wang et al., 2024b). Resistance mechanisms are further compounded by reduced membrane permeability due to porin loss (e.g., CarO mutations), overexpression of efflux pumps (e.g., AdeABC), and alterations of penicillin-binding proteins (PBPs) (Macesic et al., 2025). Mobile genetic elements such as plasmids and integrons accelerate the dissemination of resistance genes (Abdul-Mutakabbir et al., 2021). Recent reports also highlight increasing resistance to polymyxins and tigecycline, further limiting treatment options (Chen et al., 2024). Novel agents, such as sulbactam-durlobactam and cefiderocol, show therapeutic promise but face challenges with MBL-producing strains (Kollef et al., 2023).

### 4.3 Diagnostic methods and innovations

Current diagnostic approaches for CRGNB can be broadly categorized into three main strategies: phenotypic detection, molecular genotyping, and synergistic antimicrobial testing. Phenotypic methods, particularly the modified carbapenem inactivation method (mCIM) and its ethylene diamine tetraacetic acid (EDTA)-modified version (eCIM), continue to be the cornerstone of phenotypic assays due to their cost-effectiveness, operational simplicity, and reliable performance (Zhang et al., 2022; Gill et al., 2020). These assays are especially valuable in resource-limited settings where molecular techniques are unavailable. However, their inability to identify specific resistance mechanisms limits their utility for targeted therapy.

Molecular diagnostics have revolutionized CRGNB detection by enabling the rapid identification of resistance genes. Polymerase chain reaction (PCR)-based assays, such as Xpert Carba-R Assay (Cepheid, Sunnyvale, CA, USA), which target key carbapenemase genes (such as *bla*_KPC_, *bla*_NDM_, *bla*_VIM_, *bla*_IMP_, and *bla*_OXA-48_), deliver results within 1 to 4 hours. This rapid diagnostic method significantly shortens the time to appropriate therapy, ultimately improving patient survival rates (Xu et al., 2024). WGS offers the most comprehensive resistance profiling, including plasmid analysis and mutation detection. However, it remains primarily a research tool due to its complexity and high cost (Liu et al., 2023). Emerging technologies show great promise for improving diagnostic speed and accuracy. These include artificial intelligence (AI)-assisted matrix assisted laser desorption ionization-time of flight (MALDI-TOF) mass spectrometry (MS) and clustered regularly interspaced short palindromic repeats (CRISPR)-based detection systems (Cao et al., 2024). The rapid rise of AI in CRGNB diagnostics is driven by the limitations of traditional phenotypic methods (e.g., mCIM and eCIM) in detecting mixed resistance mechanisms, such as the coexistence of carbapenemase production and porin gene mutations, and by the growing need for rapid turnaround in critical care settings, where delayed targeted therapy can significantly increase mortality. For instance, machine learning algorithms integrated with MALDI-TOF MS have successfully predicted antibiotic resistance patterns in *K. pneumoniae*, enabling early and precise treatment planning (Yaşar-Duman and Çilli, 2021). Moreover, CRISPR and CRISPR-associated protein (Cas) systems, enabling rapid resistance gene detection (<2 hours), hold significant diagnostic potential (Liang et al., 2023). This shift from “retrospective resistance confirmation” to “prospective treatment guidance” allows AI models to predict resistance phenotypes in under 2 hours by integrating data such as MS, clinical metadata, and genomic signatures. This approach is particularly useful for MBL-producing strains. Future AI tools designed for point-of-care use in resource-limited settings could help bridge the diagnostic gap between high-income and low- and middle-income countries where CRGNB epidemics are accelerating. However, challenges in standardizing these tools, along with high costs, constrain their widespread adoption in resource-limited settings.

Synergy testing methods, including checkerboard assays and time-kill studies, play a crucial role in evaluating antibiotic combinations against CRGNB, particularly MBL-producing strains (Hattori et al., 2013; Brennan-Krohn and Kirby, 2019b). These methods assess fractional inhibitory concentration (FIC) indices or bactericidal kinetics, identifying effective antibiotic combinations. Automated checkerboard assays are increasingly enhancing screening efficiency (Brennan-Krohn and Kirby, 2019a). Furthermore, recent innovations include computational tools like MultiSyn, a deep learning-based platform that integrates multi-source biological data—including molecular structures, genomic profiles, and pharmacological properties—to systematically predict synergistic antibiotic combinations (Jin et al., 2025). By combining these tools with rapid diagnostics and AMR data, clinicians will be able to make timely and informed treatment decisions. Therefore, in future, efforts should focus on standardizing protocols, improving accessibility, and addressing emerging antibiotic resistance to optimize the management of CRGNB.

### 4.4 Therapeutic drugs and innovative approaches

Our bibliometric data highlight a gradual shift in the treatment paradigm, moving from last-resort agents (such as polymyxins and tigecycline) to novel β-lactam/β-lactamase inhibitor combinations. Ceftazidime-avibactam has become a first-line therapy for KPC and OXA-48-producing CRE, while meropenem-vaborbactam and imipenem-relebactam provide alternative treatment options for KPC producers (Karaiskos et al., 2025). It is noteworthy that the antimicrobial spectrum of imipenem-relebactam encompasses KPC-2/KPC-3 mutant subtypes (Papp-Wallace et al., 2023). Aztreonam-avibactam, or the combination of ceftazidime-avibactam with aztreonam, offers an effective strategy against MBL-producing strains by exploiting aztreonam’s stability against MBLs (Taha et al., 2023). At the same time, aztreonam-avibactam demonstrates a high level of sensitivity to CRKP strains that produce KPC-31, KPC-33, KPC-49 and KPC-94 (Ding et al., 2023). For CRPA, particularly difficult-to-treat resistant *P. aeruginosa* (DTR-PA), ceftolozane-tazobactam demonstrates consistent efficacy against strains harboring common resistance mechanisms (Salem et al., 2025). Ceftazidime-avibactam and imipenem-relebactam can also be utilized as first-line therapies for DTR-PA. Notably, the antibacterial activity of imipenem-relebactam remains unaffected by various porin mutations in DTR-PA (Freed and Hanson, 2024). Furthermore, a study found that levofloxacin-induced MexS gene mutation, which causes secondary resistance to imipenem-relebactam in KPC-producing *P. aeruginosa* infections (Wang et al., 2024a). The recent approval of sulbactam-durlobactam represents a significant therapeutic advancement. It specifically target OXA-23 carbapenemases in CRAB, addressing a critical unmet medical need in antimicrobial therapy (Miller et al., 2024). Eravacycline serves as a valuable option for CRGNB infections, particularly for CRAB, due to its enhanced stability against common tetracycline resistance mechanisms (Zhong et al., 2025). Nevertheless, it is imperative to acknowledge the emergence of eravacycline heteroresistance in CRAB, which cannot be disregarded (Li et al., 2024). Furthermore, eravacycline remains ineffective against CRPA. Cefiderocol represents a major therapeutic breakthrough with its unique siderophore mechanism, which confers activity against all carbapenemase classes (Ferrara et al., 2025); however, emerging resistance, particularly in MBL-producing strains, has been observed (Li et al., 2024). Antimicrobial agent synergy testing can be used to evaluate the *in vitro* activity of antimicrobial drug combinations against CRGNB when monotherapy is ineffective. However, the selection of antimicrobial agents for treating CRGNB infections is limited, as these newly approved antimicrobial agents have not yet been included in routine antimicrobial susceptibility testing.

Non-antibiotic therapies for CRGNB still face challenges, but phage therapy using bacteriophages to selectively kill pathogens has emerged as a promising strategy to tackle two major issues in the field. First, the exhaustion of the antibiotic pipeline is a growing public concern. Since 2020, only three new antibiotics (e.g., cefiderocol, sulbactam-durlobactam, and imipenem-relebactam) have been approved for CRGNB, which all show limited efficacy against MBL-producing CRAB and CRPA (Kollef et al., 2023). Phages circumvent traditional resistance mechanisms, such as efflux pumps and porin loss, by binding specifically to bacterial cell walls, making them effective against XDR and PDR strains. Second, there is an increasing need for targeted therapy. Unlike broad-spectrum antibiotics, which disrupt the gut microbiota and can promote secondary CRGNB colonization, phages exhibit narrow host specificity, minimizing collateral damage to commensal bacteria (Zurabov et al., 2023). Phage therapy has demonstrated efficacy in case studies against CRAB and CRPA; however, scalability and regulatory hurdles remain significant challenges for the therapy itself (Zurabov et al., 2023; Hayakawa et al., 2025). Future efforts will likely focus on overcoming these challenges, such as constructing standardized phage banks and navigating regulatory barriers, which are essential for translating case study successes into routine clinical practice. Phage-antibiotic synergy, such as phages disrupting biofilms to enhance antibiotic penetration, has emerged as a promising strategy to re-sensitize resistant strains, offering a cost-effective alternative to developing new antibiotics.

Additionally, antimicrobial peptides (e.g., endolysins) can disrupt biofilms, but struggles with stability and delivery persist (Baindara, 2025). Antibody-drug conjugates target specific pathogens while minimizing collateral damage; however, strain diversity complicates their development (Colombo et al., 2024). Efflux pump inhibitors (e.g., NV716) restore antibiotic susceptibility by blocking bacterial resistance mechanisms, yet concerns over toxicity remain (Wang et al., 2021). Immunomodulatory approaches, including vaccines and cytokine therapies, aim to enhance host defenses. However, this endeavor is accompanied by several challenges, including lack of a comprehensive grasp of the factors involved in protection and the dearth of predictive animal models (Sauvat et al., 2024). Microbiota-based therapies, such as fecal transplants, have shown mixed success in decolonizing CRGNB (Merrick et al., 2025). While these strategies offer alternatives to traditional antibiotics, clinical validation, manufacturing scalability, and combining these approaches with existing drugs remain key challenges. Further research is needed to optimize their use in combating multidrug-resistant infections.

Complementing these strategies, artificial intelligence is playing an increasingly pivotal role in therapeutic development. Deep learning methods have been utilized to construct predictive networks for antimicrobial activity, accelerating the discovery of novel antibiotics (Stokes et al., 2020). The integration of AI with microbiology and clinical medicine holds promise for identifying synergistic drug combinations and optimizing treatment regimens; however, external validation of these models remains a critical need.

### 4.5 Environmental dimensions and infection control

Keyword cluster 14 (hospital sewage) emphasizes the “One Health” concept, marking a critical paradigm shift in the understanding of CRGNB. This emerging perspective underscores that CRGNB is no longer seen solely as a clinical issue but as a cross-boundary threat affecting human, animal, and environmental ecosystems. This shift is driven by accumulating evidence that environmental reservoirs—such as hospital sewage, agricultural runoff, and animal populations—play a key role in the spread of resistance genes through mobile genetic elements (MGEs) like plasmids, integrons, and transposons (Klümper et al., 2024). For example, the *bla_NDM_* gene, commonly detected in sewage systems, can be transferred horizontally among *Enterobacteriaceae* via IncX3-type plasmids, contributing to community-acquired CRE infections, especially in regions with limited healthcare infrastructure (Yang et al., 2024). These plasmids can also transfer horizontally to commensal *Enterobacteriaceae*, establishing a “silent reservoir”—a hidden source of resistance genes— for community-acquired CRGNB infections. Furthermore, the emergence of zoonotic CRGNB strains, such as TMexCD1-TOprJ1-positive K. pneumoniae from livestock, which harbor plasmid-mediated RND-type tigecycline resistance determinants that reduce tigecycline efficacy in infection models, underscores the urgent need for integrated surveillance across veterinary, environmental, and clinical domains (Lv et al., 2020). Traditional, single-sector monitoring systems fail to capture these cross-boundary transmission routes.

The convergence of virulence and resistance genes further complicates containment efforts. Studies have shown that genetic exchange between hvKP and CRE can lead to the emergence of high-risk clones with pandemic potential, characterized by increased virulence and multiple resistance (Wang et al., 2025a). This phenomenon is particularly prevalent in Asia but is increasingly being reported globally, emphasizing the role of international travel and trade in the spread of resistance (Potter et al., 2016).

Addressing this complex challenge requires a multifaceted approach. Experts emphasize the urgent need for: (1) enhanced integrated surveillance systems that bridge clinical and environmental monitoring; (2) innovative wastewater treatment technologies targeting resistant pathogens; and (3) robust AMR programs (Singh et al., 2025). It is highlighted that only through coordinated international collaboration can we hope to contain the ecological spread of CRGNB, which continues to evolve at a pace that threatens to outpace existing therapeutic and preventive strategies.

## 5 Limitations

Although this study strictly adhered to standard bibliometric methods and used multiple databases to produce systematic research results, the following limitations still apply. We focused solely on the WoSCC, Scopus, and PubMed databases, while excluding alternative databases such as Google Scholar. Furthermore, limiting the analysis to only English-language literature may lead to an underestimation of the global impact of academic research conducted in non-English-speaking countries, as these findings may not have been adequately considered. Additionally, due to the subjective nature of interpreting the figures, slight differences exist between the analysis results and the actual results.

## 6 Conclusion

The global challenge posed by CRGNB demands continuous and comprehensive efforts across diagnosis, treatment, prevention, and control. Future research is likely to focus on the following key areas: (1) developing novel antimicrobials targeting carbapenemases, biofilms, and efflux pumps, as well as exploring alternative non-antibiotic therapies; (2) advancing rapid diagnostic technologies, such as CRISPR-based systems and AI-assisted tools, for the rapid identification of drug-resistant genes; and (3) disrupting transmission pathways through genomic epidemiology and One Health strategies. Despite these promising directions, several critical obstacles remain, including the rapid evolution of resistance genes, the expansion of environmental and agricultural reservoirs, and significant disparities in diagnostic and therapeutic access across regions. Overcoming these challenges requires enhanced international collaboration to promote knowledge exchange, ensure equitable resource distribution, and drive multidisciplinary innovation. Specific actions could include establishing multinational funding programs or creating global data-sharing platforms to strengthen international cooperation. By uniting global research efforts, we can curb the escalating threat of CRGNB and protect public health for future generations worldwide.

## Data Availability

The raw data supporting the conclusions of this article will be made available by the authors, without undue reservation.
